# Rachel Challice's Vow

**Published:** 1888-02-25

**Authors:** Lina Nevill

**Affiliations:** Author of "A Future on Trust," "Juno," &c.


### Rachel Challice's Vow.

By Lina Nevill, Author of "A Future on Trust," "Juno," &c.

### Chapter. X.—A Surprise.

"Yet still methinks some condescending power
Ranged the ideas in my mind that hour."
—*Thomson.*

The season, with its whirling tide of gaiety, had waned and vanished. Goodwood had robbed London of its upper ten. The Park was deserted, the Row boasted but a sprinkling of horses. Every day cabs and railway omnibuses, crowded with inmates and piled with miscellaneous luggage, made their way towards the different London stations.

Porters were driven wild under the press of work, and lost their heads as cab after cab rolled up and stood in lines three or four deep, while frantic demands for their services met their ears on every side.

Would-be travellers sat disconsolate on their luggage, waiting for the necessary assistance that was not forthcoming, and grew desperate as they saw the hands of the clock veer round towards the hour of departure.

The line of ticket-takers rivalled the stream outside the pit door of a theatre on a popular first night.

How they were got off and sorted into their right trains was a mystery, and reflected infinite credit on the overworked, passenger-assailed officials.

Sea-side lodging-house keepers were reaping their annual harvest; while the visitors endured every sort of discomfort and unpleasantness in order to do what was considered the correct thing.

The children enjoyed it to be sure. *They* did not mind sleeping in rickety beds, washing out of cracked basins, or eating their food with forks of dubious lustre and knives of undoubted bluntness. *They* did not object to breakfasting, dining, teaing and supping, and finally sitting in one and the self-same room; or take exception to the horse-hair sofa, the crochet antimacassars, or drawing-room suite of walnut wood and green rep.

To meet strange fellow-lodgers at every turn of the stairs was a joy to *them.* So long as they were allowed to roll and dig to their hearts' content in the sand, bathe every day, fish with a crooked pin in the pools on the rocks, eat as much as they wanted, with a supplement of unlimited buns and biscuits between meals, the discomforts of sea-side lodgings were nothing to them.

In London the houses were shuttered, and blinds closely drawn. Cats ranged boldly round the area railings, and cabs took the first fares that offered.

Parliament rose, and the desertion became more complete. No one with any pretention to fashion allowed him or her self to be seen in London; or, if they did, they bemoaned the hardship with loud and bitter lamentation.

Grosvenor Square was one among the many deserted regions. But there was still one house in the Square where the blinds were only lowered to keep out the sun, and the shutters closed but at night. The flowers in the window-boxes were changed as constantly, and kept as fresh and bright as in the height of the season; and tradesmen's carts rattled up as punctually as ever to take the morning orders.

Nell Endsleigh was still a prisoner on her couch, and Rachel Challice was still her cheerful devoted nurse.

They had grown very fond of each other, those two girls—the rich, courted, young heiress, and the lonely hospital nurse. After all, Rachel, in years, was little more than a girl as age counts; all her sympathies were with youth. But she was old and staid in her ways. Hardships had made her thoughtful, and responsibility had ripened her mind.

In face, she looked younger than in the days of her Welsh life. She was not so thin and careworn. She had more colour in her cheeks, altogether more brightness and expression in her refined attractive face.

Nell called her lovely, and raved about her large grey eyes and the pure perfect oval of her face. Even Charley, who could see no beauty in any girl but his Nell, owned that Nurse Challice was a good-looking young woman. Higher praise Nell could not wring from him, though she tried hard to embue him with her own impulsive, warm-hearted admiration of Rachel.

Poor Nell was very dull and rather low-spirited those sultry, long-houred August days.

With unselfish persistency she had urged Charley to go as usual to his Scotch moor, and invite the friends he usually had with him on the Twelfth.

He had consented at length, his objections over-ruled by her entreaties; and in his heart, though he hated to leave Nell, he was glad to go.

Secretly, he had dreaded being left comparatively alone in London while his different friends went off, as a matter of course, to their usual autumn pursuits—some shooting, some yachting, some fishing in Norway and Ireland—till he alone, of all his particular set, was left friendless and unoccupied.

Of course there was Nell. But then he was not allowed to be with Nell the whole day, nor would she have wished it. Though his visits were her only utterly happy times, she was too unselfish to be contented to keep him for hours at a stretch sitting with her in a hot room, with the stifling August heat outside and scarcely a breath of fresh air blowing in from the baked sunburnt gardens and the dreary dusty street.

She was much better, and her doctor gave her hopes of being able to move about a little in the course of two or three weeks.

She longed to get away from London heat, and dust, and glare, to see the summer land of harvest spread out in its golden brown richness beneath the clear country sun, and feel the fresh breezes blowing straight from the moor or sea, untainted by the breath of a great city. She pined for the broad green fields, and great densely-foliaged trees, with their wide-spreading branches casting long cool shadows on the grass; to see the harvest moon, with its broad silver disc, gleaming white and clear in the blue night sky, with the million star points—night's candles— throbbing and burning around her, while the heavy dewdrops hung on the grass and flowers, and glittered like diamond stones in the white moonlight. Another thing that made her confinement doubly hard to bear was the thought of how different all would have been had she not met with her accident.

Her wedding-day had been fixed originally for the 20th of July. That day had come and gone, and found Nell, Nell Endsleigh still. After that Sir Charles and Lady Austin had planned to be at Roxferline Castle, Charley's Scotch house, by the Twelfth, ready to receive the first detachment of a long succession of visitors.

That had been the idea. The reality found Nell in Grosvenor Square, a prisoner for the next month at least, and Charley entertaining a few bachelor friends for a few days' shooting before he hurried back to deserted London and his captive Nell. It needed all the girl's natural brightness to keep up her spirits under these trials; and Rachel was her great help through all that wearing time.

Nell made the nurse her confidante in everything. She poured out to her all her petty annoyances and disappointments, and felt happier for being able to give vent to her feelings.

It was easier to talk to Rachel than to her mother; for Mrs. Endsleigh fretted over her darling's trials, and thought almost more about them than the girl herself.

Nell would even—crowning honour of all!—read Rachel little scraps out of Charley's daily letters from Perthshire.

"I am sending off some grouse, and a little basket of white heather, as you said you liked the last time;" or, "Ranger worked splendidly to-day. I shall keep him after all, as I fancy he will be the best dog of the lot."

Such scraps as these were picked out for Rachel's benefit.

Charley did not shine as a letter writer. And, as of course, all the tender passages, which contained the gist of the letters, were too sacred for any mortal ears, Rachel privately did not appreciate the honour, nor was she so much interested in knowing the number of grouse shot, or the names of the dogs and ponies used, as Nell, with her partial love for the writer, believed everyone else must necessarily be. However, Rachel feigned sufficient interest and appreciation to satisfy Nell; and was daily treated to a few bald, not over well expressed, facts culled from Sir Charles' letters.

"I don't think I could ever have survived this time if I had had Price instead of Nurse Challice," observed Nell, as she and her mother sat alone one day.

"Ah, you admit that for once I was right and you were wrong."

"I am not sure about that," replied Nell, unwilling to own the fact, whatever she may have privately thought. "The plan was a great risk, and might have failed utterly. You see it was the merest chance your card turning up the ace of trumps; it might have been the worst one in the pack."

"Well, dear, Nurse Challice having proved herself an ace, I am quite satisfied."

"She is a trump at all events!" responded the girl warmly.

"I wonder what made her take to nursing," observed Mrs. Endsleigh. "She looks like a woman with a history."

"I do not think her life has been happy always, replied Nell." "She is very reserved about her own affairs. I asked her about her family one day, and she turned it off as quickly as she could, saying she was an orphan with scarcely any relation in the world; and then she went out of the room, pretending to fetch something for me."

"I wonder she has not married, with that pretty face and pleasant manner."

"She is too much a women's woman, if you know what I mean," replied Nell laughing. "I don't believe Nurse Challice would flirt to save her life. Men would be afraid of her, with her sweet serious manner; she would be too grave for them, they would never feel at ease with her."

"You seem to know all about it, my dear," observed Mrs. Endsleigh, much amused. "Since when did you set up as a judge of what men do and do not like in a wife?"

"Since I was engaged," replied Nell demurely. "I have taken lessons from Charley, and he has told me what sort of girl is most popular in the matrimonial market; and from his description I am quite certain Nurse Challice would only be bought by a person who knew what a superior article was when he saw it."

August dragged on slowly to Nell Endsleigh; while the month flew by only too swiftly to those whose holiday days were numbered by the date. The thirty-first was a day to which Nell looked forward as eagerly as many others dreaded its advent. Charley was to come back to her on the thirty-first. That day to many holiday-makers meant return to London; a going back to the old treadmill routine of care and toil, business and money-making.

There was a great stir and preparation in the blue and white sitting-room in Grosvenor Square as seven o'clock on the thirty-first of August drew near.

Fresh flowers were in all the vases, fresh plants in the pots; and the bunch of white heather was given the place of honour on the table by Nell's couch.

Rachel was set to watch at the window, so that she might be able to announce the fact the moment she saw a hansom with the expected visitor drive into the square.

For half an hour she stood watching. Her eyes ached with trying to keep them fixed at the same moment upon Duke Street, North Audley Street, and Lower Brook Street, the three possible ways by which a cab from King's Cross might arrive; while every moment Nell kept asking her if she saw Charley coming.

"A modern edition of Sister Anne on the watch-tower!' laughed Nell at length. "My Selim seems quite as late in ariving as the original gentleman. Do you see a detachment of the Guards advancing in a cloud of dust, or any other striking feature to carry out the resemblance?"

"Nothing but a cat's-meat man doing a very small business; and a four-wheeler with the driver asleep on the box, Miss Endsleigh," replied the watcher at the window.

As she spoke, the sound of a hansom rattling out of Duke Street, and making straight for their door, came upon the ears of the two anxious listeners.

"Sister Anne! Sister Anne! what do you see?" asked Nell, eagerly.

"Your Selim," replied Rachel with a smile. "He is getting out of the hansom, and paying the driver."

"Make haste, nurse!" called Nell excitedly. "Come here directly, or I shall be too late."

A few minutes after Charley's quick footsteps were heard on the stairs. A moment later and his well-known sharp knock sounded on the door. Rachel opened it, and stood aside.

Sir Charles gave her a kindly nod, and looked eagerly towards the couch.

It was empty, and, coming towards him from the window, with slow, careful steps, he saw his Nell, once more able to stand and walk.

"Look, Charley, I am well again!" she cried out merrily, in her clear ringing voice.

A moment later she was in his arms; and Rachel passed out, quietly shutting the door behind her, and left the lovers to themselves.

*(To be continued.)*

